# Measuring customer satisfaction on the cleanliness of food premises using fuzzy conjoint analysis: A pilot test

**DOI:** 10.1371/journal.pone.0256896

**Published:** 2021-09-01

**Authors:** Sook Yee Lim, Ummilia Balqis Harun, Abdul Rahman Gobil, Noor Afiqah Mustafa, Nur Azwanis Zahid, Syafinaz Amin-Nordin, Umi Kalsom Md. Ariffin, Tengku Zetty Maztura Tengku Jamaluddin, Nurul Azmawati Mohamed, Nor Afiah Mohd Zulkefli, Shamarina Shohaimi

**Affiliations:** 1 Department of Medical Microbiology, Faculty of Medicine and Health Sciences, Universiti Putra Malaysia, Seri Kembangan, Selangor, Malaysia; 2 Faculty of Applied Sciences, UCSI University, Cheras, Wilayah Persekutuan Kuala Lumpur, Malaysia; 3 Department of Mathematical Science, Faculty of Computer and Mathematical Sciences, Universiti Teknologi MARA (UiTM) Kampus Seremban, Seremban, Malaysia; 4 Department of Computer Science, Faculty of Computer and Mathematical Sciences, Universiti Teknologi MARA (UiTM) Kampus Seremban, Seremban, Malaysia; 5 Department of Basic Medical Sciences 2, Faculty of Medicine & Health Sciences, Universiti Sains Islam Malaysia, Nilai, Negeri Sembilan, Malaysia; 6 Department of Community Health, Faculty of Medicine and Health Sciences, Universiti Putra Malaysia, Seri Kembangan, Selangor, Malaysia; 7 Department of Biology, Faculty of Science, Universiti Putra Malaysia, Seri Kembangan, Selangor, Malaysia; Univerza v Mariboru, SLOVENIA

## Abstract

Determining the level of customer satisfaction in cleanliness regarding a product or service is a significant aspect of businesses. However, the availability of feedback tools for consumers to evaluate the cleanliness of a restaurant is a crucial issue as several aspects of cleanliness need to be evaluated collectively. To overcome this issue, this study designed a survey instrument based on the standard form used for grading the food premises and transformed it into a seven Likert scale questionnaire and consists of seven questions. This study employed fuzzy conjoint analysis to measure the level of satisfaction in cleanliness in food premises. This pilot study recruited 30 students in Universiti Teknologi MARA (UiTM) Seremban 3. The student’s perception was represented by the scores calculated based on their degree of similarities and corresponding levels of satisfaction, whereby, only scores with the highest degree of similarity were selected. Furthermore, this study identified the aspects of hygiene that assessed based on the customers’ satisfaction upon visiting the premises. The results indicated that the fuzzy conjoint analysis produced a similar outcome as the statistical mean, thus, was useful for the evaluation of customer satisfaction on the cleanliness of food premises.

## Introduction

Satisfaction refers to the emotion, response, and reaction of consumers to products or services. Customer satisfaction, on the other hand, is defined as the evaluation of the customers’ experience upon receiving a product or service. Kursunluoglu (2011) described customer satisfaction as a comparison between the expectations and perceptions of the product or service performance [1]. Customer satisfaction is achieved when the perceived performance of the product or service meets or surpasses their expectations. Satisfaction is the crucial success factor in any business as satisfied customers tend to repurchase the product in the future and recommend it to others. According to Zairi (2000), customers became dissatisfied with the product if it did not achieve their expectations, while satisfied customers were loyal to a product or service if the product meets the customers’ expectations [2].

Many factors were identified to influence customer satisfaction when visiting food premises, including, food taste, service, price, environment and hygiene [3, 4]. A study conducted by Fatimah and colleagues (2011) on Malaysian customers found that cleanliness was the main factor followed by food variety and location of the premises as criteria in selecting dining places [5]. This observation indicated that customers have deep concerns regarding the risk of foodborne diseases. In Malaysia, food premises, including food catering, food outlets, stalls, canteens, and food trucks, are required to comply with the requirements of the Food Hygiene Regulations 2009 under the Food Act 1983. These premises must also undergo routine inspections by the Ministry of Health and local government to ensure cleanliness maintenance of the premises [6]. In short, the premises’ hygiene was found to guarantee customer satisfaction.

Customer satisfaction can be considered to determine the level of cleanliness of a premise, albeit [7] study found that customers created optimistic biases, which believed that foodservice establishments have fewer risks for pathogens. However, Wang and colleagues (2020) claimed that customers possessed excellent self-protection behaviour due to their high level of awareness regarding hygiene and health while they pursued appetising food [8]. The customer’s insight may also be used to improve the particular system in a large scale, for instance, taking the assessment of customers’ perception into account to increase Canada’s food safety system [9].

Studies in the United States (US) found that personal hygiene of food handlers was a significant factor contributing to customer satisfaction and their intention to revisit despite some areas required improvements, such as table hygiene, food temperature, and handwashing lavatory [10]. However, studies regarding the satisfaction of Malaysian customer were only focused on ambience, food, and service quality provided by the food premises [11–15]. Correspondingly, due to their acquired knowledge in foodborne diseases, university students were suited as the respondents in the study associated with food and premises hygiene [16].

The food service should be enhanced to meet the customers’ expectations since they are willing to purchase the food at these premises. Therefore, food operators should practice a food safety culture during food handling, as customers are willing to pay a premium to reduce food-related casualty risks at premises [17]. Food operators will inevitably incur significant losses if they ignore these factors because dismayed customers tend to avoid revisiting food premises with foodborne disease outbreaks [18].

Measuring satisfaction can be done by several methods. Many researches used statistical such as percentage, mean and standard deviation as a medium to evaluate customers’ satisfaction level but this method is not exactly accurate. For instance, according to Rasmani and Shahari (2007) they concluded that evaluation of job satisfaction by using percentage cannot produce consistent result [19]. Another method which is Fuzzy conjoint analysis, has been used in measuring job satisfaction, student’s perception, satisfaction on credit card service but has yet to be tested in food industry area, is widely used nowadays to evaluate customer satisfaction because this method is closest approach to customer’s decisions. This method involves Likert scale to represent linguistic terms. In this study, the questionnaire is being redesign from dichotomous method which evaluate by enforcer that give answer whether yes or no to Likert scale. Therefore, the customers can also take part in evaluating the performance of the food premises. Hence, this study focused on the attributes that evaluate the cleanliness of food premises by university students using fuzzy conjoint analysis.

### Background of theory

This section consists of a brief review of the fuzzy conjoint analysis method, which evaluates the level of satisfaction by employing a fuzzy set of preferences that represent the values of attributes evaluated by the respondents. Commonly, “statistical significance” is used as a primary criterion to determine whether the hypotheses are either accepted or rejected, which is inherently less informative and lack of sensitivity to detect the significant effect. Meanwhile, the fuzzy set theories were developed to deal with imprecision and uncertainty, which helps the researchers to decide the appropriate samples for further studies based on the necessity instead of just merely rejecting the null hypothesis [20].

The expectations of the respondents are essentially ambiguous, unclear, and have a subjective character based on personal feelings. The fuzzy set theory preferably combines with conjoint analysis due to the subjective uncertainty and imprecise importance of preferences, particularly in estimating the relative importance of the selected attributes [21].

The term fuzzy conjoint analysis was introduced by Turksen and Willson (1995) to analyse consumer preferences and is applied in various studies by employing the Likert scale to represent the linguistic terms evaluated [22]. The Likert-type scale is used to represent the linguistic terms that measure satisfaction. In this study, the 7-point Likert scale was used to measure the levels of satisfaction ranging from ‘7: very strongly agree’ to ‘1: very strongly disagree’. The 7-point Likert scale was recommended for surveys related to satisfaction being more robust and sensitive in reflecting the respondent’s real evaluation with minimum interpolation. This type of scale also perceived accuracy and can be efficiently utilised by the researcher [23].

In total, seven user satisfaction levels corresponding to seven fuzzy linguistic values were created based on the 7-point Likert scale [19]. By using this method, the response provided by each respondent was compared with the response from respondents evaluated in the study. In addition, each linguistic value had its own fuzzy set. Since each linguistic value obtained a degree of membership, issues on determining the importance of an item were unlikely to occur. From a broader perspective, the fuzzy conjoint method using linguistic values is an appropriate analysis tool for data containing a fuzzy set such as the level of satisfaction.

The conjoint analysis is a survey-based technique that helps marketers measure the consumers’ value of a product or service. According to Rasmani and Shahari (2007), this conjoint analysis is known as a ‘trade-off’ analysis that is widely used in marketing research [19]. There are several types of conjoint analysis such as the menu-based conjoint analysis by Orme [24], adaptive choice-based conjoint analysis by DeSarbo and colleagues [25], choice-based conjoint analysis by Cunningham and colleagues [26], and fuzzy conjoint analysis by Rasmani and Shahari [19]. In this study, the fuzzy conjoint analysis was used to measure the satisfaction of consumers regarding the cleanliness of food premises. This method was previously proposed by Turksen and Willson [22]. The fuzzy set, R, represented the values of criterion evaluated by the respondents. The degree of membership for each element, *y*_*j*_ in the fuzzy set, R, was defined as follows:

$$\mu_{R}(y_{j},A)=\sum_{i=1}^{n}[\frac{w_{i}}{\sum w_{i}}].\mu_{F_{i}}(x_{j},A)$$

Where,

$\frac{w_{i}}{\sum w_{i}}$ = Weight that represented the level of satisfaction for respondent *i* concerning other respondents

*μF*_*i*_(*x*_*j*_,*A*) = Membership value degree for the respondent *i* for item A according to the linguistic label *x*_*j*_ = 1,2….,*t*

*A* = an item/a question

*n* = number of linguistic labels

**Fig 1:**
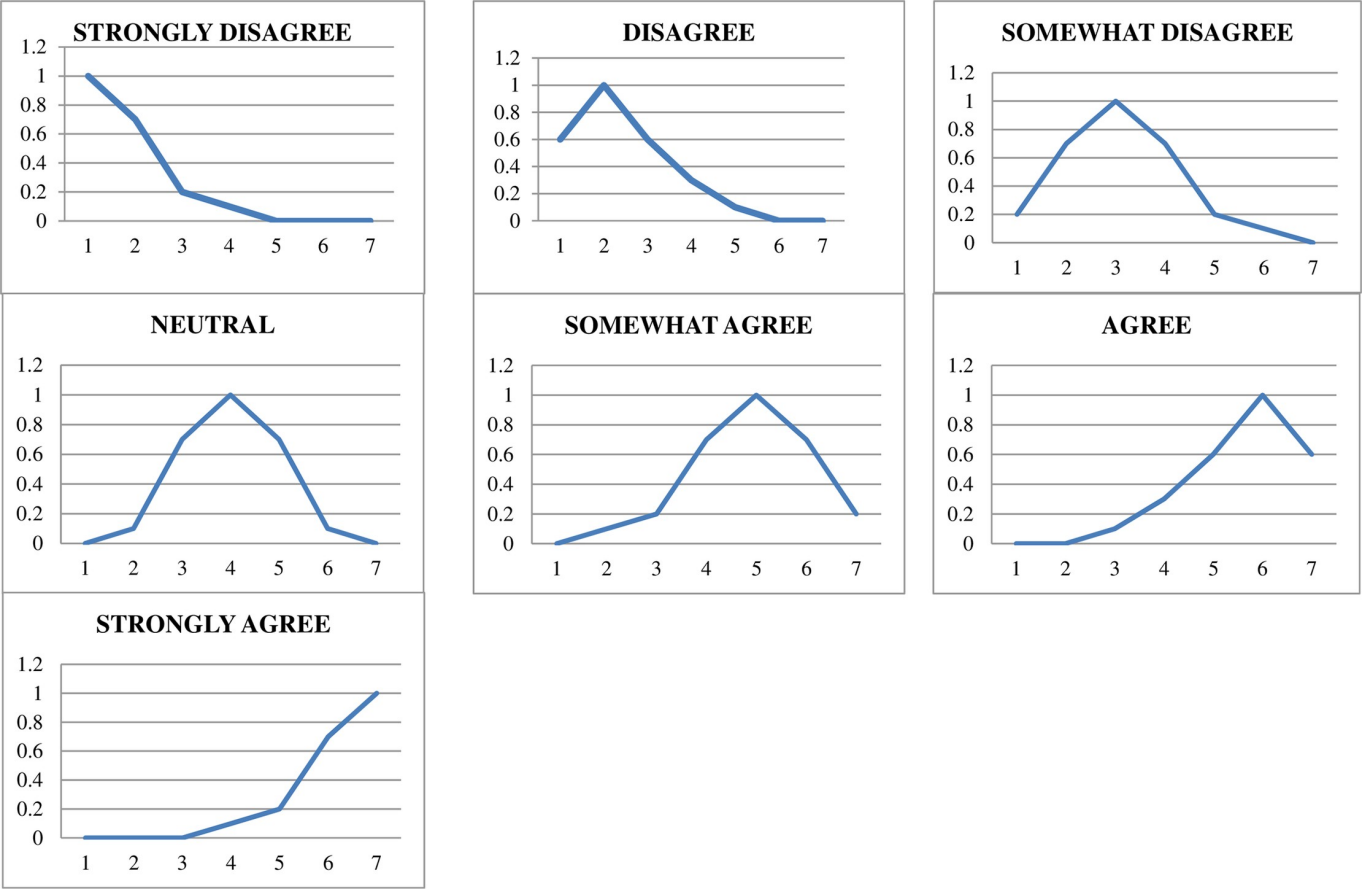
Graph of the fuzzy linguistic value.

The application of linguistic variables was introduced by Zadeh [27]. In this study, seven linguistic variables adapted from Rasmani and Shahari (2007) were used and defined as follows (Table 1) [19]. Fig 1 shows the visualisation by using the graph of the fuzzy linguistic value for the 7-point Likert scale.

**Table 1:** Fuzzy linguistic value representing each agreement level.

Rating value	Linguistic Variables	Fuzzy Linguistic Value
1	Strongly Disagree	{11,0.72,0.23,0.14,05,06,07}
2	Disagree	{0.61,12,0.63,0.34,0.15,06,07}
3	Somewhat Disagree	{0.21,0.72,13,0.74,0.25,0.16,07}
4	Neutral	{01,0.12,0.73,14,0.75,0.16,07}
5	Somewhat Agree	{01,0.12,0.23,0.74,15,0.76,0.27}
6	Agree	{01,02,0.13,0.34,0.65,16,0.67}
7	Strongly Agree	{01,02,03,0.14,0.25,0.76,17}

Adapted from Rasmani and Shahari (2007).

The membership value degree was calculated based on the formula described above, then, compared with the fuzzy linguistic variables. This step was performed using the fuzzy similarity measure based on the formula of dot product based on the Euclidean inner products defined below:

$$S\left(F,M\right)=\frac{F•M}{\max(F•F,M•M)}$$

where,

*F* = (*μ*_*R*_(*x*_1_),*μ*_*R*_(*x*_2_),…) *M* = (*μ*_*M*_(*x*_1_),*μ*_*M*_(*x*_2_),…) F, M are vectors,

*X* = (*x*_1_,*x*_2_,…)

By using the fuzzy similarity formula, the similarity values for each level of satisfaction were determined. The outcomes of this analysis were determined based on the maximum similarity value obtained among the satisfaction levels.

## Method

### Study design and participants

A survey was conducted on 30 randomly selected students who aged 18 and above and were from Faculty of Computer and Mathematical Science (FSKM), Faculty of Sports Science and Recreation (FSR) and Faculty of Administrative Science & Policy Studies (FSPPP) of UiTM Negeri Sembilan branch (Seremban campus), Malaysia. This survey took place at a restaurant nearby the campus from 10 am to 12pm at 14th March 2020. The survey was conducted using Google Form because it is the easiest and efficient method to collect the data. Moreover, according to the Statista (2021), more than six billion people use smartphones worldwide in 2021, and this number is predicted to rise in coming years [28]. The study protocol was approved by Universiti Sains Islam Malaysia Research Ethics Committee with the reference number—USIM/JKEP/2019-61. A written consent was obtained before the participant started the survey.

### Survey instrument

**Fig 2:**
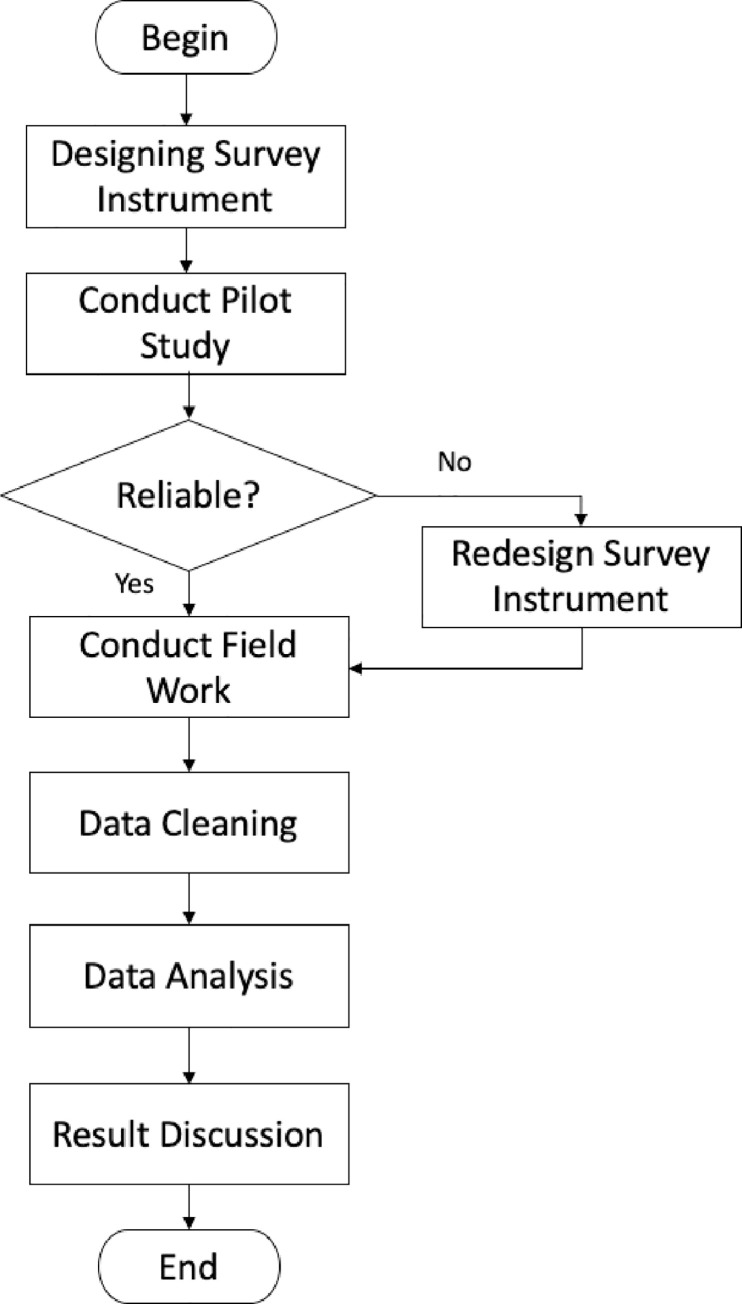
The framework of the study process.

This study adapted the method used by Rasmani and Shahari (2007) [19]. Hence this chapter will discuss the process as depicted in Fig 2. The first stage in this study was to design the survey instrument. The survey instrument was adapted from the “borang pemeriksaan dan pengredan permis makanan”. This form was developed by the Ministry of Urban Wellbeing, Housing and Local Government, and is officially used by local authorities during the inspection and grading the food premises. The study selected 29 questions that were suitable for consumers to be included in the survey instrument.

In the second stage, this study was conducted on a small scale to understand the objectives of the study, which ensured that the utilised instruments met the requirements of the study and that the questionnaire could be clearly understood by the respondents [29]. The questionnaire was converted from a dichotomous answer to Likert scale pattern. After the pilot survey was conducted, a Cronbach’s alpha was performed to check the reliability of the questionnaire after the data is collected.

Upon the second stage’s completion, the questionnaire used was redesigned and sent to the medical expert, public health, and microbiologist for the vetting process. After the vetting process, seven questions were included in the questionnaire. Then, the reliability of the questionnaire was tested again.

The responses were analysed using statistical mean and fuzzy conjoint analysis. In total, seven questions from the questionnaire were discussed by experts. Items in the questionnaire corresponded to several criteria which included the following:

1. The entire store, including the floor, wall, and ceilings are clean and odourless (A1).
2. All utensils and equipment in each area, including the wiping cloth, are clean (A2).
3. Food handlers to appear in full attires (apron, head cover, gloves, and covered shoes) (A3).
4. Food handlers do not sneeze or cough while preparing food (A4).
5. Pests (cockroaches, flies, or rats) are seen (A5).
6. Food handlers do not smoke while serving food (A6).
7. The food is well covered (A7).

## Data analysis and results

Table 2 shows the characteristics respondents. The mean age of respondents was 21 years old and close to 97% were females. More than 75% of respondents were degree students and more than half of respondents was from mathematics field of study.

**Table 2:** Demographic background of the students (n = 30).

Characteristics	Mean ± SD	Frequency	Percentage
Age	20.6 ±1.89		
Gender			
Male		1	3.3%
Female		29	96.7%
Level of study			
Diploma		7	23.33%
Degree		23	76.67%
Field of study			
Mathematics		19	63.33%
Administration		8	26.67%
Sports		3	10.0%

Table 3 presents the output of the SPSS analysis. Based on Table 3, a reliability analysis performed on the perceived task values scale comprising seven items. Cronbach’s alpha value indicated that the questionnaire reached the acceptable reliability, α = 0.751. Most items appeared to be worthy of retention, resulting in a decrease in the alpha if deleted. The one exception to this was item A5, which would increase the alpha to α = 0.775. However, the differences are negligible hence A5 was retained.

**Table 3:** Item analysis from SPSS output.

Scale Statistics		Mean	Variance	Std. Deviation 4.204	N
		22.39	17.670		7
Item-total Statistic	Scale Mean if Item Deleted	Scale Variance if Item Deleted	Corrected Item-Total Correlation	Squared Multiple Correlation	Cronbach’s Alpha if Item Deleted
A1	18.84	15.564	0.323	0.429	0.748
A2	18.95	13.944	0.640	0.583	0.698
A3	19.54	12.467	0.554	0.490	0.700
A4	19.18	13.183	0.580	0.558	0.697
A5	19.70	14.249	0.256	0.161	0.775
A6	18.63	13.594	0.402	0.380	0.736
A7	19.47	11.647	0.635	0.568	0.677
		Alpha	Standardise item alpha		
Reliability Coefficients		0.751	0.766		

The survey was conducted to study the satisfaction levels of customers regarding the cleanliness of food premises. Each item was measured using a 7-point Likert scale representing different levels of satisfaction. Table 4 below presents the distribution score.

**Table 4:** Distribution scores for restaurant cleanliness.

Statements	Strongly disagree (%)	Disagree (%)	Somewhat disagree (%)	Neutral (%)	Somewhat agree (%)	Agree (%)	Strongly agree (%)
A1	0%	3.33%	20.0%	33.33%	23.33%	13.33%	6.67%
A2	3.33%	3.33%	16.67%	36.67%	36.67%	3.33%	0%
A3	20.0%	10.0%	26.67%	26.67%	13.33%	0%	3.33%
A4	0%	0%	26.67%	30.0%	36.67%	6.67%	0%
A5	16.67%	6.67%	23.33%	13.33%	26.67%	13.33%	0%
A6	3.33%	3.33%	6.67%	16.67%	30.0%	20.0%	20.0%
A7	3.33%	10.0%	16.67%	36.67%	16.67%	16.67%	0%

Table 5 demonstrates the comparison of decisions obtained from the customers’ satisfaction levels on the cleanliness of food premises based on two types of evaluation methods, namely, the fuzzy conjoint method, and the statistical mean. The overall result presented in Table 5 indicated that the result obtained using mean are consistent with the result obtained using fuzzy conjoint for A2 and A7 while for others the result will differ by one-scale.

**Table 5:** Comparison of methods used to evaluate the levels of customer satisfaction on the cleanliness of food premises.

Items	Methods	Strongly Disagree	Disagree	Somewhat Disagree	Neutral	Somewhat Agree	Agree	Strongly Agree	Level of Satisfaction
A1	Mean	4.4333							Neutral
	Fuzzy Conjoint	0.1964	0.3852	0.569	0.7268	0.7624	0.6911	0.5161	Somewhat Agree
A2	Mean	4.1							Neutral
	Fuzzy Conjoint	0.2146	0.4441	0.6536	0.8489	0.8315	0.6667	0.3919	Neutral
A3	Mean	3.1667							Somewhat Disagree
	Fuzzy Conjoint	0.2227	0.447	0.6505	0.8294	0.8243	0.6808	0.4268	Neutral
A4	Mean	4.2333							Neutral
	Fuzzy Conjoint	0.3745	0.4737	0.7275	0.8513	0.8755	0.6682	0.576	Somewhat Agree
A5	Mean	3.6667							Neutral
	Fuzzy Conjoint	0.2776	0.4479	0.5916	0.715	0.7596	0.68	0.4852	Somewhat Agree
A6	Mean	5.0667							Somewhat Agree
	Fuzzy Conjoint	0.1141	0.2397	0.388	0.5585	0.7395	0.8029	0.7154	Agree
A7	Mean	4.0333							Neutral
	Fuzzy Conjoint	0.2376	0.4352	0.6182	0.7651	0.7639	0.6551	0.4516	Neutral

Using fuzzy conjoint method, customers are ‘Strongly Agree’ with question A6 and ‘Agree’ with question A1, A4 and A5. Meanwhile, the results of analysis using mean showed that the employers ‘Agree’ only for question A6. Additionally, there are no customer ‘Disagree’ for all questions from fuzzy conjoint result, but by using mean, customers are ‘Disagree’ for question A3 which describe on the food handlers attire.

Based on Table 5, the analysis on *All utensils and equipment at each area including wiping cloth are clean* (A2) and *All food is well covered* (A7) using fuzzy conjoint are consistent with the result using mean which customers’ level of satisfaction is ‘Moderate”. Meanwhile, the result obtained by using standard deviation for A2 is 1.0619 which showed that the customers’ satisfaction level is between ‘Disagree’ and ‘Agree’, similar with A7.

However, the analysis on *The entire store including the floor*, *wall and ceilings are clean and odorless* (A1), *Food handlers do not sneeze or cough while preparing food* (A4) and *Pests (cockroaches*, *flies or rats) are seen* (A5) showed that customers ‘Agree’ by using fuzzy conjoint method but customers felt ‘Moderate’ using mean value. By using standard deviation, for A1, the satisfaction level is distributed between ‘Disagree’ and ‘Strongly Agree’, for A4, the dispersion of customers’ satisfaction level is between ‘Disagree’ and ‘Agree’ and, for A5, the level of satisfaction dispersed from ‘Strongly Disagree’ until ‘Agree’.

Further, for question A3, customers’ satisfaction is ‘Moderate’ from fuzzy conjoint result, but customers are ‘Disagree’ using mean value. Based on standard deviation result, customers’ satisfaction level scattered from ‘Strongly Disagree’ to ‘Agree’. Lastly, for question A6, using fuzzy conjoint, customers are ‘Strongly Agree’ but ‘Agree’ by using mean. As for the standard deviation result, the level of satisfaction spread from ‘Disagree’ until ‘Very Strongly Agree’. Therefore, the result obtained from fuzzy conjoint method consistent for two questions and slightly different for the other question.

## Discussion

About 20.0% of the students chose ‘strongly agreed’ on the criteria that the restaurants’ food handlers should not smoke while serving food as the main criteria to ensure their satisfaction with the service provided. Meanwhile, another 20.0% chose ‘strongly disagree’ for the criteria that suggested food handlers to appear in full attires (apron, head cover, gloves, and covered shoes), which may guarantee them to revisit the premise given their satisfaction.

The overall results presented in Table 5 depicted the comparison of satisfaction levels based on the two types of evaluation methods used, namely, the statistical mean and fuzzy conjoint analysis. The results obtained using mean consistent with the results obtained using fuzzy conjoint analysis method. For instance, customers chose ‘somewhat agree’ for questions A1 (The entire store, including the floor, wall, and ceilings are clean and odourless) A4 (Food handlers do not sneeze or cough while preparing food) and A5 (Pests (cockroaches, flies, or rats) are seen) after being analysed by the conjoint analysis but only ‘neutral’ based on the mean value. Cleanliness is the practice of cleaning a surface, including floors by using equipment such as brushes or sweepers and soaps. In addition to being cost-effective, cleanliness was associated with the aesthetic aspects that pleased the customers, in which food operators frequently devoted their attention to satisfy, attract and create the customers’ loyalty. Although the practice is an excellent effort to attract customers, it still needs improvement. For example, besides using soap for cleaning, the food handler may also implement a food safety culture by using bleach that has antimicrobial properties, which ensures that premises are not only clean but also pathogen-free [30].

Sneezing or coughing while preparing food is strictly prohibited due to the possibility of food contamination. The lack of handwashing after covering the mouth or nose during sneezing and coughing may also contribute to the risk of pathogen trans-mission in food. Therefore, food handlers are encouraged to sanitise their hands after sneezing or coughing with hand sanitiser after using water and soap to eliminate pathogens. To ensure effective handwashing, the usage of hand sanitisers in the form of gel or foam is recommended as it can easily be absorbed, moisturising, no sticky texture, cleaner, and are less smelly [31]. However, customers also chose ‘somewhat agree’ that pests were seen at the restaurants visited. Pest manifestation in the premise is an unhealthy indicator, which the restaurant operator needs to find ways to keep their premises free from any pests. Flies are among the most common pests in food premises, which may contaminate food if left uncontrolled. Specifically, flies are identified as a vector for foodborne pathogens such as Enterobacteriaceae, Escherichia coli and Staphylococcus aureus, which also possess antibiotic-resistant profile [32]. One of the most common methods to eliminate flies is through the use of insecticides, although it is not widely recommended due to the toxic effects on other organisms [33]. Therefore, food premises operator should practice regular and simpler sanitation that is cost-effective compared to other methods, which, in effect, controls the fly population [34].

On the other hand, for question A3 (Food handlers to appear in full attires (apron, head cover, gloves, and covered shoes)), the customer’s response was ‘neutral’ based on the fuzzy conjoint analysis, while customers selected ‘strongly disagree’ using the mean value. This result indicated that the attires worn by food handlers when handling food were presumably unsuitable to the customers’ expectations. Notably, the food handler’s attires have the potential to transmit food pathogens if it is not in a hygienic condition. Therefore, Malaysian food handlers are advised to comply with the Food Act 1983 and the Food Hygiene Regulations 2009, which requires them to wear clean and bright clothing comprising an apron, head covering, and shoes. Additionally, they should not wear accessories like jewellery and watches to avoid any cross-contamination risk, with some food handlers, for instance, use gloves when dicing vegetables to prevent this risk. This idea is an excellent practice, albeit precautions should be taken as gloves usage without proper handwashing may instead increase the risk of bacterial contamination [35].

Additionally, for question A2 (All utensils and equipment in each area, including the wiping cloth, are clean) and A7 (The food is well covered), the fuzzy conjoint analysis indicated a ‘neutral’ level of customer satisfaction, which aligned with the obtained results using statistical mean. Therefore, food handlers need to enhance their efforts in improving both aspects to meet customer satisfaction. Maintaining the cleanliness of cooking utensils and wiping cloth used is another way to prevent cross-contamination. For example, food handlers should not use the same wiping cloth for different cleaning purposes to ensure that there is no risk of pathogen transmission from one surface to another. Apart from the use of different wiping cloths, the type of material used to make the cloth should also be taken into account in dealing with the spread of pathogens. Cloths made from microfiber are better for cleaning compared to cotton as this material is capable of significantly minimising the transmission of bacterial spores during the surface cleaning [36]. The use of food covers is also crucial to protect cooked or raw food from pests such as flies, which are known as a highly mobile pest. The flies can fly from the filth to the food before secreting their saliva that contains digestive enzymes and harmful pathogens [37], which may cause pathogen contaminated food if left exposed.

Using the fuzzy conjoint method, customers selected ‘agree’ and ‘somewhat agree’ when analysed using mean for question A6 (Food handlers do not smoke while serving food). Generally, a food handler should not smoke during the entire food handling period since the risk of food contamination by tobacco smoke may occur, which may cause the tobacco smoke to remain on clothes or the smoker’s hands known as third-hand smoking [38]. The burning tobacco releases hazardous chemicals, such as lead, cadmium, mercury, and arsenic instead of nicotine that may harm a human’s health [39]. Therefore, food handlers are required to practice personal hygiene, including handwashing and wearing clean attire while handling food so that customers are confident with the services provided.

Based on the analysis made based on the mean value, most items showed ‘neutral’ response (A1, A2, A4, A5, and A7) compared to conjoint analysis, which indicated a ‘somewhat agree’ response. Responses analysed using conjoint analysis indicated customer’s opinion and its attributes. This research finding indicated that conjoint analysis provided an accurate result for customer satisfaction than the mean based approach. Additionally, the analysis can offer a more objective perspective in emphasising studies that need subjective assessment related to satisfaction and service due to its realistic simulation on customer’s circumstance [40].

Based on the results obtained from this study, it can be concluded that fuzzy linguistic values can be used to differentiate the various levels of customer satisfaction. A particular strength of this study is that the use of fuzzy value is appropriate, which involves subjective evaluation or rating because the Likert scale is considered as a discrete integer value. The integer value can often reflect actual differences between categories of scale [41]. However, some limitations of this study should be mentioned, namely, the sample size, being inconsiderably big enough to generalise the findings.

Some limitations of this study are worth noting. First, this study involved a small sample size and non-random sampling technique, which may affect the findings’ generalizability. However, with the consideration of pilot testing, the main focus of this study was not looking for the generalization but for the usability of the fuzzy conjoint analysis to measure the level of cleanliness in food premises. Second, the respondents answered the questionnaire at different times. As a result, different rate was given by each respondent even though the questionnaire was answered in the same day. Furthermore, the type of survey instruments used in this study which was a questionnaire in Google form can tend to be bias. It is because the respondents just going through to answer the questionnaire and some of the questions may not be answered.

## Conclusion

This study presented that fuzzy conjoint analysis can be an alternative method to evaluate customer satisfaction on the cleanliness of food premises. The findings showed that the application of fuzzy conjoint analysis can be used to evaluate customer’s satisfaction on cleanliness of food premises. This study can provide useful information to other researchers especially in food health industry. For instance, the fuzzy conjoint analysis can be used by restaurant owners to identify which aspects of hygiene are most crucial to their customers when visiting food premises. Furthermore, this analysis is also practical and may widely be used in conducting studies based on the customer’s behaviour involving their decision-making situation [42].

## Abbreviations

Fuzzy conjoint: also known as ‘trade-off’ analysis and widely used in marketing research
Cronbach Alpha: a measure of internal consistency
Likert-scale: a type of rating scale used to measure attitudes or opinions
Linguistic value: a value used to represent the agreement or satisfaction level
Dichotomous: involving two completely opposing ideas or things
